# Food intake, physical activity and body composition of adolescents and young adults: data from Brazilian Study of Nutrition and Health

**DOI:** 10.1186/s12889-021-11171-3

**Published:** 2021-06-12

**Authors:** Ana Paula Wolf Tasca Del’Arco, Agatha Nogueira Previdelli, Gerson Ferrari, Mauro Fisberg

**Affiliations:** 1 Pediatrics Department, Paulista School of Medicine, Federal University of São Paulo – UNIFESP, São Paulo, SP Brazil; 2 Department of Nutrition, School of Public Health, University of São Paulo – USP, São Paulo, SP Brazil; 3 Universidad de Santiago de Chile (USACH), Escuela de Ciencias de la Actividad Física, el Deporte y la Salud, Santiago, Chile; 4 Pensi Institute – José Luiz Egydio Setúbal Foundation, Sabará Children’s Hospital, São Paulo, SP Brazil

**Keywords:** Public health, Youth, Physical activity, Food intake, Anthropometry

## Abstract

**Background:**

Lifestyle acquired in youth can determine the individual’s health. Constant vigilances in all aspects related to the health of the young population is essential, and evaluate their health parameters is important. The objective of this study was to describe and to compare food intake, physical activity (PA) practice, nutritional status and body composition between adolescents and young adults.

**Methods:**

Four hundred seventy-six individuals from the Brazilian Study of Nutrition and Health (EBANS) were analyzed. Food intake was evaluated by applying two 24-h Dietary Recall. The PA and sitting time (ST) were measured by the International Physical Activity Questionnaire in minutes/week and metabolic equivalent task (METs). Body weight and waist circumference (WC) were measured. Body mass index (BMI) and waist circumference to height ratio (WHtR) were calculated. Mann-Whitney and Chi-Square tests were used.

**Results:**

Energy and macronutrients intake, number of meals, and breakfast skippers weren’t different between age groups. 48% of adolescents and 53% of young adults didn’t meet the PA recommendation, and adolescents practiced more PA than young adults (total PA: *p* = 0.006; METs: *p* < 0.001; leisure PA: *p* = 0.001); the individuals who studied practiced more PA (total PA: *p* = 0.034; METs: *p* = 0.029; leisure PA: *p* < 0.001) and had ST significantly higher (*p* = 0.009) than those who worked. Almost 30% of adolescents and 45% of young adults had excess weight; presenting difference according to nutritional status, WC and WHtR (*p* < 0.001).

**Conclusion:**

There is a high prevalence of excess weight among young Brazilians and differences were observed between age groups regarding nutritional status, body composition and PA practice.

**Trial registration:**

ClinicalTrials.Gov NCT02226627. Retrospectively registered on August 27, 2014.

## Background

The transition period between childhood and adulthood is very dynamic and characterized by intense bio psychosocial changes [1–3], with different age cut-off points to define it. According to the World Health Organization (WHO), adolescents are individuals between 15 and 19 years [4] and youth comprise individuals between 15 and 24 years [3, 4]. However, WHO recognizes that the terms “adolescents,” “young adults,” and “youth” are used interchangeably [4]. In the context of health aspects, this stage of life establishes some distinct age classifications, for example to assess nutritional status and adiposity [5–8], for the physical activity (PA) recommendation [9, 10], and to determine nutrient intake needs [11]. Considering this group’s current social and lifestyle behaviors, added to peculiar and dynamic biological conditions, a more comprehensive age classification for adolescence has been discussed in the literature, classifying individuals aged 10–24 years [12].

Whatever the nomenclature used to define this stage of life, the lifestyle acquired in this period can determine the individual’s health and life condition, since the choices made can have an impact on future possibilities [3, 12, 13]. In this period, health risks are diverse and rapidly changing [13, 14], in addition to biological growth and development, which are still occurring intensively and are often neglected by health systems and society [3].

Over the years, individuals’ lifestyle and health are being impacted by several factors and/or different facets of transition to adulthood [3, 12]. In adolescence, important changes can occur in the dietary behavior of individuals [13]. In Brazil, is observed an increase in the consumption of foods positively associated with the obesity, as well the replacement of traditional meals (such as rice and beans) by ultra-processed foods, even among adolescents, which may indicate a change in the Brazilian’s dietary behavior [15–17]. Also, the sedentary behavior is increasing among the adolescents around the world [10] what seems to be the consequence experienced by young people in the face of current socio-cultural changes, including the exaggerated appreciation of sedentary behavior, especially screen time (including electronic games, computer, tablet, smartphone) [18] that competes with active leisure time, culminating in a more inactive lifestyle [19].

The adoption of a healthy lifestyle, including a balanced diet and regular PA practice, should be encouraged from childhood, adolescence and throughout youth, since it affects immediately in health promotion and risk reduction that could impact the health of future generations [3, 13, 20, 21]. Several lifestyle factors are risk factors associated with cardiometabolic risk in the early stages of life [22]. Constant vigilances in all aspects related to the health and well-being of the young population is essential, since this generation is being influenced by “unprecedented global forces” [3]. In view of this fact, it is necessary to evaluate the health parameters of the young population that can be a consequence of habits and lifestyles acquired amidst the multiple behaviors inherent to this population. This study aims to describe food intake, PA practice, nutritional status and body composition of young Brazilians and to compare these parameters between adolescents (15–19.9 years) and young adults (20–24.9 years) of the Brazilian Study of Nutrition and Health (*Estudo Brasileiro de Nutrição e Saúde,* EBANS).

## Methods

### Study design

EBANS is a cross-sectional study and representative of the Brazilian population aged 15 to 65 years (*n* = 2000). It comprises all regions of the country, with sample calculation stratified by sex, age group and socioeconomic level (SEL). The Ethics Committee of the Federal University of São Paulo has approved this study, and all participants signed the Free and Informed Consent and/or the Consent Form [23]. EBANS data were collected from October, 2014 to July, 2015. EBANS is the Brazilian appendix of the Latin American Study of Nutrition and Health (*Estudio Latinoamericano de Nutrición y Salud,* ELANS), a multicenter urban area study conducted in eight Latin American countries [24].

**Fig. 1:**
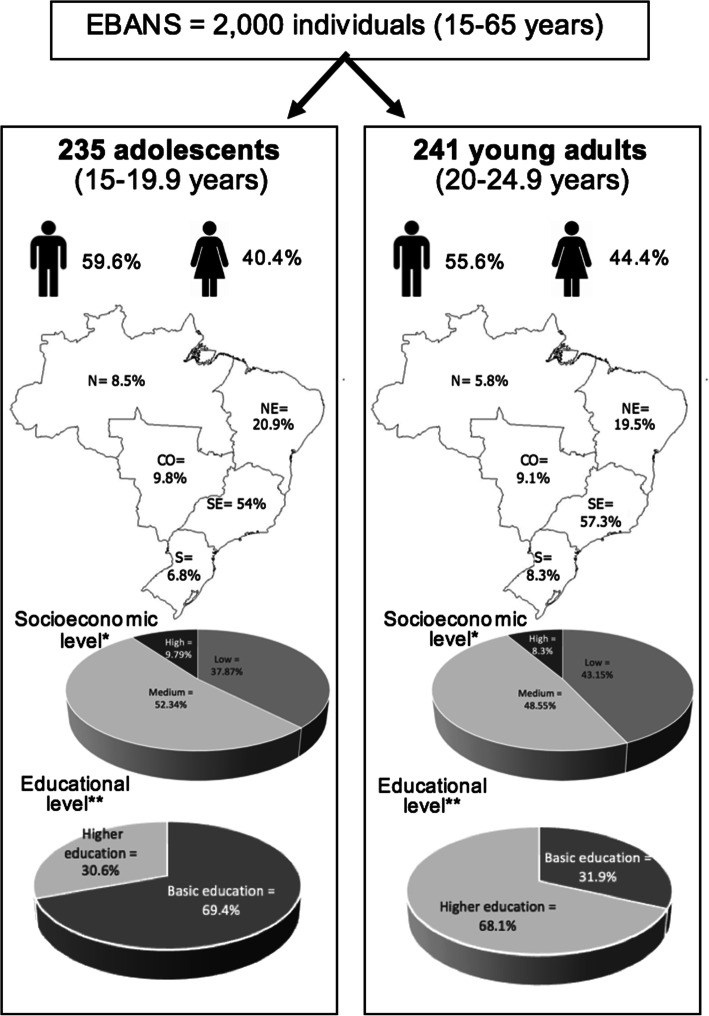
Sociodemographic characterization of the target population. N: North region; NE: Northeast region; CO: Midwest region; SE: Southeast region; S: South region. *Socioeconomic level: classified in three levels: high (classes A1, A2, B1), medium (classes B2, C1) and low (classes C2, D, E) [25]. **Educational level: categorized into basic education (up to high school) and higher education (complete or incomplete)

Within the sample universe of EBANS, the present study analyzed a subpopulation of 476 individuals aged 15–24.9 years. The characterization of the target population of this study is described in Fig. 1. Previously published [23], the sample selection methodology was systematic (to select urban conglomerates and households within each sampling unit) and random to select the sampling units defined based on the cartographic division of the census sectors [26]. Exclusion criteria were individuals with diseases that could affect food intake and/or energy expenditure; with physical or mental disabilities; unable to read; pregnant or lactating women; absent from home or who refused to conduct the second interview; those who didn’t live in “homes” (such as hospitals and/or orphanages); and adolescents bellow 15 years old, once they are biologically vulnerable and would be necessary to check their stage of pubertal maturation (because it affect the nutritional status and body composition), what would be impractical from a logistic point of view to a population-based study as EBANS. For all data collection, the methodology was previously published [23, 24].

### Socio-demographic characteristics

The socio-demographic characteristics, such as age, sex, region and educational level were self-reported by the participants. The educational level was categorized into basic education (up to high school) and higher education, which could be “complete or incomplete”, since 18-year-old individuals could already attend higher education, but have not completed this educational stage, which lasts an average of 4 years in Brazil. The SEL was determined according to the Standard Economic Classification Criteria Brazil [25], classified into three levels: high (grouping classes A1, A2, B1), medium (classes B2, C1) and low (classes C2, D, E).

### Food intake

Briefly, the food intake data was performed by the application of two 24-h Dietary Recall (24HR), in two home visits with an interval of 7 days; with the application of the Multiple Pass Method (MPM) [27]. 24HR has been used as the preferred methodology in several population-based studies worldwide [28], since it ensures better adherence by the interviewee because it is quick and requires less memory time for the interviewees [29], being able to provide information about the food consumption of the previous day or the last 24 h and, when replicated in the same individual, can estimate the individual’s usual nutrient and/or energy intake, considering intrapersonal variability, using a statistical modeling technique, o Multiple Source Method (MSM) [30, 31]. The data were analyzed through the Nutrition Data System for Research software version 2013 (NDS-R University of Minnesota, MN, USA); and adjusted according to intrapersonal variability through the MSM [30, 31] to estimate the usual intake of each nutrient. Two variables related to dietary behavior (number of meals eaten and breakfast skippers) were analyzed, and the energy intake and the macronutrients intake (carbohydrates, proteins and lipids).

### Physical activity

The PA and sitting time (ST) data was obtained by the application of the long-form International Physical Activity Questionnaire (IPAQ), having the last 7 days as reference. This questionnaire has been validated internationally using CSA accelerometer (model 7164) to assess total PA in individuals from 12 countries with Spearman’s correlation coefficients ranging from 0.46 to 0.96 [32]. The PA sections of transportation and leisure were included due to the greater relevance of these practices to guide public health programs in Latin American urban environments [33]. The transportation PA (walking and bicycle), and the leisure PA (walking and moderate-to-vigorous physical activities (MVPA)) were analyzed separately, according to the scoring protocol established by IPAQ (https://sites.google.com/site/theipaq/scoring-protocol). The intensity of 3.3 metabolic equivalent task (METs) for walking was attributed, 4.0 METs for moderate activity, and 8.0 METs for vigorous activity [34, 35]. Individuals who met the international PA recommendations [9, 10] were classified as “active”. ST included sedentary activities (time spent watching TV and DVDs, the use of other screens and reading), and it was determined by the equation: [ST (min/day) during weekdays × 5] + [weekend ST (min/day) × 2] / 7.

### Body composition

The following parameters were used to determine the body composition: body weight, the Body Mass Index (BMI), waist circumference (WC) measurement and the waist circumference to height ratio (WHtR). Data of body weight and height were measured with the aid of a Sanny® portable scale and portable stadiometer, respectively. The classification of anthropometric nutritional status was determined based on the BMI according to the parameters established by WHO for individuals > 19 years [6], and according to gender and age for adolescents [5]; and it reflects the “weight status”. WC was measured with Sanny® inelastic tape according to WHO recommendations [36], and categorized based on reference data for adolescents according to sex [37]. WC > 88 cm for women and > 102 cm for men was used as a cut-off bridge for determining central obesity in young adults [38]. The WHtR was also calculated, and was classified as inadequate when ≥0.54 for adolescents [7] and ≥ 0.5 for young adults [8].

### Statistical analyses

Descriptive statistics was used for the categorical variables and, for the continuous, a 95% confidence interval, the measures of central tendency, and the 25th, 50th and 75th percentiles were calculated. Using the Willet, Howe and Kushi [39] method, the food intake data were adjusted by energy. The Kolmogorov-Smirnov test pointed out heterogeneous data distribution and the Mann-Whitney test was used to compare energy intake, macronutrients intake and PA level and ST, WC and WHtR (continuous variables) between the age groups. For the comparison of categorical variables (BMI, type of occupation, number of meals and breakfast skippers), the Chi-Square test was used. For all statistical tests, a 5% significance level was used. The statistical analyses were performed by Stata software (Statistics/Data Analysis, version 13.0, Texas, USA) and measured considering the sample weight [24].

## Results

The total number of participants included in this study was 476 (50.6% young adults); and the mean age of the adolescents was 17.06 years (±1.44), and young adults, 22.11 years (±1.43). In overall, about half of the population was from the Southeast region, male and classified as medium by SEL. Furthermore, around 70% of the adolescents had basic education while the same proportion of the young adults had higher education. Figure 1 illustrates the characterization of the population. Regarding type of occupation, the majority of adolescents were students (63.2%), 22.7% had work jobs, 10.7% were unemployed and 3.4% had household functions. Among young adults, more than half were working (57.6%), 21.6% were unemployed, 13.6% were students and 7.2% had household functions. A statistical difference between age groups was found (*p* < 0.001).

Regarding the dietary behavior, it was verified that the mean number of meals was four in both age groups: 4.13 (95%CI 3.98–4.29) among adolescents and 4.03 (95%CI 3.88–4.18) among young adults. Also, that the minority of adolescents (12.8%) and young adults (7.5%) omitted breakfast (did not have the meal in either 24HR), and more than half of the adolescents (66.8%) and young adults (68.5%) consumed breakfast habitually (reported in both 24HR). There were no statistical differences between both age groups (*p* = 0.131).

Energy and macronutrients intake were not different between both age groups (Table 1). The caloric distribution of macronutrients was 51.7 and 51.7% for carbohydrates, 17.1 and 17.5% for proteins, and 31.2 and 30.8% for lipids, for adolescents and young adults, respectively.

**Table 1:** Energy and macronutrients intake, according to age groups

Nutrients	Age group	n	Percentiles			95%CI	***p*** value*
			25	50	75		
Energy intake (kcal)	15–19.9	235	1504.54	1963.91	2410.32	1947.66–2117.64	0.2728
	20–24.9	241	1555.58	1900.61	2213.73	1893.38–2057.15	
Carbohydrates (grams)	15–19.9	235	197.57	253.54	310.40	251.96–275.10	0.1518
	20–24.9	241	194.52	237.14	286.85	241.66–262.98	
Proteins (grams)	15–19.9	235	66.23	82.54	99.77	81.59–88.70	0.6287
	20–24.9	241	65.38	81.37	101.33	80.65–88.52	
Lipids (grams)	15–19.9	235	50.58	66.80	85.61	67.26–73.64	0.1199
	20–24.9	241	51.05	64.07	79.26	63.75–69.65	

*Mann-Whitney at the 5% significance level

*95%CI* 95% confidence interval

Significantly, adolescents practiced more total PA (METs-min/week and min/week) and leisure (min/week) than young adults. Overall, adolescents did 860 METs-min/week and 100 min/week more than young adults. For transportation PA, no statistical difference was found between both groups. In total, 48.1% of adolescents and 52.7% of young adults did not comply with the WHO recommendation for PA (*p* = 0.206). Regarding ST, there was no statistical difference between both groups (Table 2). PA data were also evaluated according to the type of occupation of individuals (considering only individuals who worked and studied). We found that those who worked practiced less PA than those who studied, significantly for total PA time (min/week), for total METs-min/week, and for leisure PA, except for transportation PA. The ST was significantly shorter among those who worked (Table 3).

**Table 2:** Physical activity (total and by domain) and sitting time practiced, according to age groups

Physical activity (PA)	Age group	n	Percentiles			95%CI	***p*** value*
			25	50	75		
METs-min/week	15–19.9	235	198	672	2383.50	1480.04–2176.74	< 0.001
	20–24.9	241	132	396	1059	737.58–1198.60	
Total PA (min/week)	15–19.9	235	75	250	840	362.93–458.93	0.006
	20–24.9	241	60	140	530	266.24–351.36	
Transportation PA (min/week)	15–19.9	235	30	100	200	144.60–205.57	0.359
	20–24.9	241	25	80	190	146.30–211.18	
Leisure PA (min/week)	15–19.9	235	0	60	600	244.96–341.24	0.001
	20–24.9	241	0	0	200	132.84–205.00	
Sitting time (min/week)	15–19.9	235	150	252.86	342.86	248.29–292.04	0.123
	20–24.9	241	115.71	214.29	360	232.94–281.08	

*Mann-Whitney at the 5% significance level

*95%CI* 95% confidence interval, *METs* Metabolic equivalent task, *min* minutes, *PA* Physical activity

**Table 3:** Physical activity and sitting time, according to the type of occupation

Physical activity (PA)	Type of occupation	n	Percentiles			95%CI	***p*** value*
			25	50	75		
METs-min/week	Works	191	198	462	1410.5	870.57–1457.27	0.029
	Student	180	198	672	2400	1438.68–2242.46	
Total PA (min/week)	Works	191	60	160	585	276.15–373.28	0.034
	Student	180	60	267	857.50	367.37–479.11	
Transportation PA (min/week)	Works	191	30	100	210	146.57–216.49	0.125
	Student	180	20	75	180	120.94–188.67	
Leisure PA (min/week)	Works	191	0	0	260	141.97–227.63	< 0.001
	Student	180	0	112.50	720	270.58–383.17	
Sitting time (min/week)	Works	191	115.71	214.29	334.29	223.93–276.94	0.009
	Student	180	180	270	377.14	261.64–312.30	

*Mann-Whitney at the 5% significance level

*95%CI* 95% confidence interval, *METs* Metabolic equivalent task, *min* Minutes, *PA* Physical activity

**Fig. 2:**
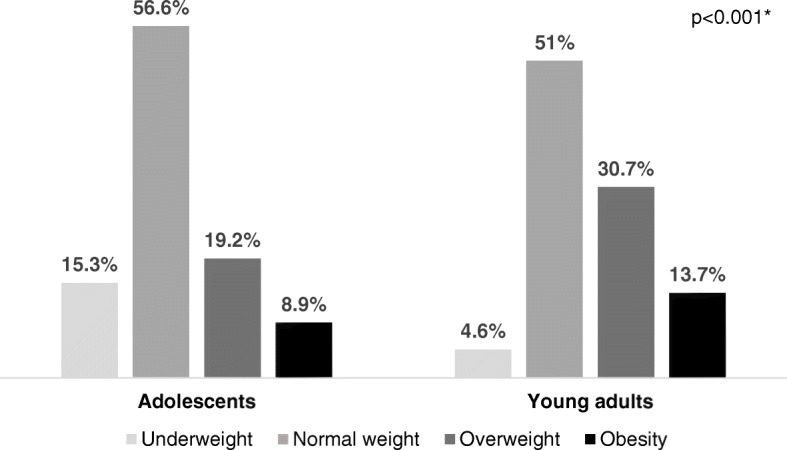
Distribution of the nutritional status classification according to the body mass index (BMI), for each age group. *Chi-square test at the 5% significance level. Nutritional status classification for adolescents: underweight = BMI for age < − 2SD; normal = −2SD ≥ BMI for age ≤ 1SD; overweight = 1SD ≥ BMI for age ≤ 2SD; obesity = BMI for age > 2SD [5]. Nutritional status classification for young adults: underweight = BMI < 18.5 kg/m^2^; normal = BMI between 18.5 and 24.9 kg/m^2^; overweight = BMI between 25 and 29.9 kg/m^2^; obesity = BMI ≥ 30.0 kg/m^2^ [6]

Almost 30% of adolescents and 45% of young adults had excess weight (Fig. 2). There was a statistical difference between both groups in relation to the classification of the nutritional status and the continuous BMI value (*p* < 0.001). Regarding the WC measurement, the median found was 74.80 cm (95%CI 75.13–78.42) for adolescents and 80.00 cm (95%CI 80.27–83.78) for young adults; and the WHtR median was 0.44 (95%CI 0.45–0.47) for adolescents and 0.48 (95%CI 0.48–0.50) for young adults. A statistical difference between groups was found for both the WC and WHtR (*p* < 0.001). The adjustment values of the measurement of WC and WHtR are different between the age groups. It was found that 13.2% of adolescents and 32.4% of young adults had inadequate WC values. The WHtR was inadequate for 17% of adolescents and 39.4% of young adults.

## Discussion

The present study evaluated EBANS individuals aged 15 to 24.9 years, divided into two age groups, which were compared in relation to nutritional status, body composition, food intake and PA practice. In general, it was found that adolescents practiced more time of PA and had lower prevalence of excess weight when compared to young adults, with no significant differences to quantitative energy and macronutrients intake. It was also found that those who worked were those who practiced less time of PA compared to those who studied, which is related to the lifestyle of individuals.

The lifestyle and nutritional status of an individual in the early stages of life require vigilance, since they can determine the health condition that will be established in adulthood, since they are associated with cardiometabolic risk already in the childhood [22, 40]; and may add psychosocial consequences if weight variation (mainly excess weight) occurs during adolescence [41]; affecting the socioeconomic context of the environment. The transition phase to adulthood is marked by significant biological and socioeconomic changes, which can expand or limit the individual’s future possibilities, according to the choices made [3, 12], either in the social and/or biological context.

The global epidemic of obesity, added to the current global syndemic of obesity, undernutrition, and climate change [42], is associated with increased risk of chronic non-communicable diseases (NCDs) [21] is the result of a combination of several factors, including food consumption and PA practice which are modifiable risk factors [40, 43]. In the present study it was found that some parameters of body composition and nutritional status of age groups were statistically different, with a higher prevalence of inadequate WC, WHtR, and excess weight among young adults. Although the cross-sectional design of EBANS does not allow a cause and effect relationship or an analysis over time, this leads to reflection on possible changes in the lifestyle of young adults that could affect their nutritional status.

The eating habits acquired in the early stages of life can have repercussions on future habits. The stimulation of healthy habits from childhood is important, since once acquired at the beginning of life the record of first learning and social forms tend to remain throughout life [43, 44]. However, substantial changes and/or discontinuity of the dietary pattern during the adolescence phase can occur [13]. In the present study, it was found that the habit of consuming breakfast was usual for most individuals; similar to data from the National Food Survey (*Inquérito Nacional de Alimentação,* INA) [45]. The National School Health Survey (*Pesquisa Nacional de Saúde do Escolar*, PeNSE) revealed that the eating breakfast habit has increased 4.5% among Brazilian adolescent students from 2012 to 2015 [46].

Comparing the caloric intake data of the adolescents of the present study with the data from INA [47] and from Adolescent Cardiovascular Risk Study (*Estudo dos Riscos Cardiovasculares em Adolescentes,* ERICA) [48], it was possible to identify a similar quantitative intake; although there are some chronological and methodological differences between the above mentioned studies and EBANS. Regarding the caloric contribution of macronutrients to the total caloric values, the values found in EBANS for the adolescents were lower than those presented in the literature [47, 48] for carbohydrates (EBANS: 51.7%; ERICA: 54%; INA: 57%); slightly higher for proteins (EBANS: 17.1%; ERICA and INA: 16%); and similar to ERICA data for lipids (EBANS: 31.2%; ERICA: 31%; INA: 27.5%). According to DRIs [49], the caloric distribution of macronutrients was quantitatively adequate among adolescents in the present study and, for young adults, according to WHO [50], the caloric distribution reached the upper limit for lipids, was lower for carbohydrates and higher for proteins.

Regarding the sufficiency of PA practice, it was found that approximately half of adolescents and young adults did not meet the recommendations for PA practice [9, 10]; however, there was no statistical difference between individuals classified as active in both age groups, since the WHO recommends twice the time of PA to be practiced by individuals aged 5–17 years compared to those over 18 years old. According to data from the three editions of PeNSE [46, 51, 52] there was a higher prevalence of active adolescents in the present study. Comparing with data from the Surveillance of Risk Factors and Protection for Chronic Diseases by Telephone Survey (*Vigilância de Fatores de Risco e Proteção para Doenças Crônicas por Inquérito Telefônico,* VIGITEL) 2019 [53], which evaluated young people aged 18–24 years, a higher prevalence of young people who did not meet the PA practice recommendations was found in the present study.

The literature has reported that the PA practice in the early stages of life can increase the probability of individuals being active in the subsequent stages [20, 43]. The data from the present study showed that adolescents have been practicing more PA than young adults, both in METs-min/week and in total PA and leisure time; however, it should be noted that the EBANS design does not allow the population to be assessed and/or compared over time. Physical inactivity during leisure time is associated with increased adiposity in adults, assessed independently or in conjunction with lack of total PA [54]. In the present study, excess weight and adiposity were more prevalent among young adults, together with a higher prevalence of physical inactivity during leisure time. The encouragement of PA practices during leisure time should be the focus of public policies, with reorganization of urban spaces to adapt public spaces where leisure and recreation activities could be performed [55].

The type of occupation was determinant for the PA practice, that is, those who have been working practiced less total PA and leisure. It was found that the median PA leisure time of young adults was 0 min/week; as well as the median PA leisure time of the individuals who worked (0 min/week). Behavior that can be associated with lifestyle, since assuming an economically active role in society, linked with social transitions, acquisition of responsibilities, and affirmation of individual identity, can impair habits and behaviors [12]. Entering into the labor market, one of these social transitions assumed by young people, may affect behavioral and lifestyle aspects. Young adults represent an economically important group for society; since they are considered the healthiest stratum of any population [2]. Their nutritional status can also affect cognitive and executive functions, impairing the developed work activities [56], as well as even influencing even remuneration [41].

PA practice during transportation is an opportunity to increase the PA practice of those who have been working. In the present study, it was found that transportation PA was not statistically different among age groups nor according to the type of occupation. Active transportation should be encouraged with public policies that facilitate urban mobility and allow the transportation of the population through non-motorized means of transport, such as bicycles, which requires safe and wide bike lanes [55].

Sedentary behavior is related to the individuals’ lifestyle. Although there are no parameters that define a sedentary behavior based on ST, and no official public health guidelines on the ST limit that can impair health, because there is no sufficiently consistent scientific data basis for this [57], studies have reported that frequent interruptions in ST can positively impact some cardiovascular parameters [58]. In the present study, it was found that the ST was significantly shorter among those who worked. This finding may be associated with the profile of the activity developed by those who start in the labor market, since the search for the first job is marked by insertion difficulties. This is more prominent for the most qualified functions [59], which can be the activities performed by those who enter the labor market, less sedentary activities with more dynamic and multiple functions.

In view of the above, the data lead to the reflection that a new behavior seems to be assumed by young adults, mainly in relation to the PA practice, which was significantly lower among young adults. This fact could be a consequence of the adaptation to the new social roles assumed by young people entering the labor market, impacting their habits as well as lifestyle and reducing the PA practice, since the individuals who have been working were the ones who practiced less PA.

The main limitations of this study refer to possible overestimations and/or underestimations of the data reported by the interviewees through the questionnaires; however, the main strength of EBANS is the data collection methodology, with the additional techniques to minimize possible errors and biases [23], added to the rigorous training of the professionals who collected the data. The use of the same methodology, scientifically validated, in all regions of Brazil, including the population of adolescents and young adults, is another strength of the present study. It is important to note that EBANS was conducted in urban area, and the generalization of results for the rural area of the country should be cautious.

In view of the findings of this study, with some parameters analyzed that differed between age groups (such as PA and body composition variables), some statistical analyzes were conducted to assess possible correlations and/or associations; however, such analyzes were not statistically significant.

## Conclusions

In conclusion, the findings of the present study show a high prevalence of excess weight among young Brazilians, being significantly higher among young adults. The energy and macronutrients intake was similar between age groups. The time of PA practice was significantly longer among adolescents; however, the percentage of those who did not meet the PA recommendations wasn’t different between age groups. The differences in health parameters between adolescents and young adults should be considered in the elaboration of specific actions related to improving the health and quality of life of the young population. It’s important to emphasize that the age group from 15 to 24.9 years is too broad and heterogeneous. It presents important differences in the multiplicity of behaviors assumed by young people as a result of the non-uniformity of the process chosen for the responsibilities acquisition.

Such findings can be a consequence of a new social behavior assumed by young people and, in this context, consequences are observed in the health parameters of individuals. Therefore, further studies are necessary to properly investigate such behaviors and their association with health parameters. Adolescents and young people are often noticed only as a group in transit, which is detrimental to being perceived and truly considered as individuals who have rights and duties in all society settings [3].

## Data Availability

The datasets used and/or analyzed during the current study are available from the corresponding author on reasonable request.

## Abbreviations

BMI: Body Mass Index
EBANS: Brazilian Study of Nutrition and Health
ELANS: Latin American Study of Nutrition and Health
ERICA: Adolescent Cardiovascular Risk Study
INA: National Food Survey
IPAQ: International Physical Activity Questionnaire
MET: Metabolic Equivalent Task
MPM: Multiple Pass Method
MSM: Multiple Source Method
MVPA: Moderate-to-Vigorous Physical Activity
NCDs: Chronic Non-Communicable Diseases
PA: Physical Activity
PeNSE: National School Health Survey
ST: Sitting Time
VIGITEL: Surveillance of Risk Factors and Protection for Chronic Diseases by Telephone Survey
WC: Waist Circumference
WHO: World Health Organization
WHtR: Waist Circumference to Height Ratio
24HR: 24-h Dietary Recall

## References

[1] Altikulaç S, MGN B, Foulkes L, Crone EA, Van Hoorn J (2019). Age and gender effects in sensitivity to social rewards in adolescents and young adults. Front Behav Neurosci.

[2] Das JK, Salam RA, Thornburg KL, Prentice AM, Campisi S, Lassi ZS, Koletzko B, Bhutta ZA (2017). Nutrition in adolescents: physiology, metabolism, and nutritional needs. Ann N Y Acad Sci.

[3] Patton GC, Sawyer SM, Santelli JS, Ross DA, Afifi R, Allen NB, Arora M, Azzopardi P, Baldwin W, Bonell C, Kakuma R, Kennedy E, Mahon J, McGovern T, Mokdad AH, Patel V, Petroni S, Reavley N, Taiwo K, Waldfogel J, Wickremarathne D, Barroso C, Bhutta Z, Fatusi AO, Mattoo A, Diers J, Fang J, Ferguson J, Ssewamala F, Viner RM (2016). Our future: a lancet commision on adolescente health and wellbeing. Lancet..

[4] World Health Organization (1986). Young people’s healthy – a challenge for society. Report of a WHO Study Group on Young People and ‘Health for All by the Year 2000’. Technical Report Series 731.

[5] de Onis M, Onyango AW, Borghi E, Siyam A, Nishida C, Siekmann J (2007). Development of a WHO growth reference for school-aged children and adolescents. Bull World Health Organ.

[6] World Health Organization (2010). Obesity: preventing and managing the global epidemic: report of a WHO Consultation. Technical Report Series.

[7] Vasquez F, Correa-Burrows P, Blanco E, Gahagan S, Burrows R (2019). A waist-to-height ratio of 0.54 is a good predictor of metabolic syndrome in 16-year-old male and female adolescent. Pediatr Res.

[8] Ashwell M, Gibson S (2016). Waist-to-height ratio as an indicator of ‘early health risk’: simpler and more predictive than using a ‘matrix’ based on BMI and waist circumference. BMJ Open.

[9] Bull FC, Al-Ansari SS, Biddle S, Borodulin K, Buman MP, Cardon G (2020). World Health Organization 2020 guidelines on physical activity and sedentary behaviour. Br J Sports Med.

[10] Chaput JP, Willumsen J, Bull F, Chou R, Ekelund U, Firth J, Jago R, Ortega FB, Katzmarzyk PT (2020). WHO guidelines on physical activity and sedentary behaviour for children and adolescents aged 5–17 years: summary of the evidence. Int J Behav Nutr Phys.

[11] Otten JJ, Hellwig JP, Meyers LD (2006). Dietary reference intakes: the essential guide to nutrient requirements.

[12] Sawyer SM, Azzopardi PS, Wickremarathne D, Patton GC (2018). The age of adolesence. Lancet Child Adolesc Health.

[13] Poll FA, Miraglia F, D’avlia HF, Reuter CP, Mello ED (2020). Impact of intervention on nutritional status, consumption of processed foods, and quality of life of adolescents with excess weight. J Pediatr.

[14] Mokdad AH, Forouzanfar MH, Daoud F, Mokdad AA, El Bcheraoui C, Moradi-Lakeh M (2016). Global burden of diseases, injuries, and risk factors for young people’s health during 1990–2013: a systematic analysis for the global burden of disease study 2013. Lancet..

[15] Lima LR, Nascimento LM, Gomes KRO, Martins MCC, Rodrigues MTP, Frota KMG (2020). Association between ultra-processed food consumption and lipid parameters among adolescents. Cienc Saude Coletiva.

[16] Cunha DB, da Costa TH, da Veiga GV, Pereira RA, Sichieri R (2018). Ultra-processed food consumption and adiposity trajectories in a Brazilian cohort of adolescents: ELANA study. Nutr Diabetes.

[17] Monteiro CA, Levy RB, Claro RM, de Castro IR, Cannon G (2010). Increasing consumption of ultra-processed foods and likely impact on human health: evidence from Brazil. Public Health Nutr.

[18] LeBlanc AG, Gunnell KE, Prince SA, Saunders TJ, Barnes JD, Chaput JP (2017). The ubiquity of the screen: an overview of the risks and benefits of screen time in our modern world. Transl J ACSM.

[19] Silva PVC, Costa Junior AL (2011). Efeitos da atividade física para a saúde de crianças e adolescentes. Psicol Argum.

[20] Hayes G, Dowd KP, MacDonncha C, Donnelly AE (2019). Tracking of physical activity and sedentary behavior from adolescence to young adulthood: a systematic literature review. J Adolesc Health.

[21] Kassebaum NJ, Arora M, Barber RM, Bhutta ZA, Brown J, Carter A, et al. GBD 2015 DALYs and HALE collaborators. Global, regional, and national disability-adjusted life-years (DALYs) for 315 diseases and injuries and healthy life expectancy (HALE), 1990–2015: a systematic analysis for the global burden of disease study 2015. Lancet. 2016;388(10053):1603–58. 10.1016/S0140-6736(16)31460-X. 10.1016/S0140-6736(16)31460-X PMC5388857 27733283

[22] Patnode CD, Evans CV, Senger CA, Redmond N, Lin JS (2017). Behavioral counseling to promote a healthful diet and physical activity for cardiovascular disease prevention in adults without known cardiovascular disease risk factors: updated evidence report and systematic review for the us preventive services task force. JAMA..

[23] Fisberg M, Kovalskys I, Previdelli AN, Zimberg IZ, Del’Arco APWT, Ferrari GLM. Brazilian Study of Nutrition and Health (EBANS) – Brazilian data of ELANS: methodological opportunities and challenges. Rev Assoc Med Bras. 2019;65(5):669–77. 10.1590/1806-9282.65.5.669. 10.1590/1806-9282.65.5.669 31166444

[24] Fisberg M, Kovalskys I, Gómez G, Rigotti A, Cortés LY, Herrera-Cuenca M (2016). Latin America Study of Nutrition and Health (ELANS): rationale and study design. BMC Public Health.

[25] Brazilian Association of Research Companies (2013). Standard economic classification criteria Brazil.

[26] Brazilian Institute of Geography and Statistics (2010). Census 2010.

[27] Moshfegh AJ, Rhodes DG, Baer DJ, Murayi T, Clemens JC, Rumpler WV, et al. The US Department of Agriculture Automated Multiple-Pass Method reduces bias in the collection of energy intakes. Am J Clin Nutr. 2008;88(2):324–32. 10.1093/ajcn/88.2.324. 10.1093/ajcn/88.2.324 18689367

[28] Ferrari GLM, Kovalskys I, Fisberg M, Gomez G, Rigotti A, Sanabria LYA (2020). Anthropometry, dietary intake, physical activity and sitting time patterns in adolescents aged 15–17 years: an international comparison in eight Latin American countries. BMC Pediatr.

[29] Willet W (2012). Nutritional epidemiology. 3rd ed. 529 p.

[30] Harttig U, Haubrock J, Knuppel S, Boeing H (2011). The MSM program: the web-based statistics package for estimating usual dietary intake using the multiple source method. Eur J Clin Nutr.

[31] Haubrock J, Nothlings U, Volatier JL, Dekkers A, Ocke M, Hartting U (2011). Estimating usual food intake distributions by using the multiple source method in the EPIC-Potsdam calibration study. J Nutr.

[32] Craig CL, Marshall AL, Sjostrom M, Bauman AE, Booth ML, Ainsworth BE (2003). International physical activity questionnaire: 12-country reliability and validity. Med Sci Sports Exerc.

[33] Ferrari GLM, Kovalskys I, Fisberg M, Gomez G, Rigotti A, Sanabria LYC (2020). Methodological design for the assessment of physical activity and sedentary time in eight Latin American countries – the ELANS study. MethodsX..

[34] Ainsworth BE, Haskell WL, Whitt MC, Irwin ML, Swartz AM, Strath SJ (2000). Compendium of physical activities: an update of activity codes and MET intensities. Med Sci Sports Exerc.

[35] Ainsworth BE, Haskell WL, Herrmann SD, Meckes N, Bassett DR, Tudor-Locke C (2011). Compendium of physical activities: a second update of codes and MET values. Med Sci Sports Exerc.

[36] World Health Organization (2008). Waist circunference and waist-hip ratio: report on a WHO expert consultation.

[37] Katzmarzyk PT, Srinivasan SR, Chen W, Malina RM, Bouchard C, Berenson GS (2004). Body mass index, waist circumference, and clustering of cardiovascular disease risk factors in a biracial sample of children and adolescents. Pediatrics..

[38] National Institutes of Health. Clinical guidelines on the identification, evaluation, and treatment of overweight and obesity in adults – the evidence report: NIH Publication; 1998. p. 98–4083. Available from: https://www.nhlbi.nih.gov/files/docs/guidelines/ob_gdlns.pdf. [cited 2018 Nov] 9813653

[39] Willet WC, Howe GR, Kushi LH (1997). Adjustment for total energy intake in epidemiologic studies. Am J Clin Nutr.

[40] Simmonds M, Llewellyn A, Owen CG, Woolacott N (2016). Predicting adult obesity from childhood obesity: a systematic review and meta-analysis. Obes Rev.

[41] Huang CH, Chen DR (2019). Association of weight change patterns in late adolescence with young adult wage differentials: a multilevel longitudinal study. PLoS One.

[42] Swinburn BA, Kraak VI, Allender S, Atkins VJ, Baker PI, Bogard JR (2019). The global Syndemic of obesity, undernutrition, and climate change: the lancet commission report. Lancet..

[43] Brown CL, Perrin EM (2018). Obesity prevention and treatment in primary care. Acad Pediatr.

[44] Helle C, Hillesund ER, Omholt ML, Overby NC (2017). Early food for future health: a randomized controlled trial evaluating the effect of an eHealth intervention aiming to promote healthy food habits from early childhood. BMC Public Health.

[45] Pereira JL, Castro MA, Hopkins S, Gugger C, Fisberg RM, Fisberg M (2018). Prevalence of consumption and nutritional content of breakfast meal among adolescents from the Brazilian National Dietary Survey. J Pediatr.

[46] Brazilian Institute of Geography and Statistics (2016). Nacional school health survey: 2015.

[47] Veiga GV, da Costa RS, Araújo MC, Souza AM, Bezerra IN, Barbosa FS (2013). Inadequação do consumo de nutrientes entre adolescentes brasileiros. Rev Saude Publica.

[48] Souza AM, Barufaldi LA, Abreu GA, Giannini DT, de Oliveira CL, dos Santos MM (2016). ERICA: ingestão de macro e micronutrientes em adolescentes brasileiros. Rev Saude Publica.

[49] Institute of Medicine (2002). Dietary reference intakes for energy, carbohydrate, Fiber, fat, fatty acids, cholesterol, protein, and amino acids.

[50] World Health Organization (2003). Diet, nutrition and the prevention of chronic diseases: report of joint a WHO/FAO expert consultation.

[51] Hallal PC, Knuth AG, Cruz DKA, Mendes MI, Malta DC (2010). Prática de atividade física em adolescentes brasileiros. Cien Saude Colet.

[52] de Rezende LFM, Azeredo CM, Canella DS, Claro RM, de Castro IRR, Levy RB (2014). Sociodemographic and behavioral factors associated with physical activity in Brazilian adolescents. BMC Public Health.

[53] Brazil. Ministry of Health. Surveillance of Risk and Protection Factors for Chronic Diseases through Telephone Survey: VIGITEL BRASIL 2019. Brasília: 2020. Available from: http://www.crn1.org.br/wp-content/uploads/2020/04/vigitel-brasil-2019-vigilancia-fatores-risco.pdf?x53725. [cited 2020 Jun].

[54] Du H, Bennet D, Li L, Whitlock G, Guo Y, Collins R (2013). Physical activity and sedentary leisure time and their associations with BMI, waist circumference, and percentage body fat in 0.5 million adults: the China Kadoorie biobank study. Am J Clin Nutr.

[55] Guthold R, Stevens GA, Riley LM, Bull FC (2018). Worldwide trends in insufficient physical activity from 2001 to 2016: a pooled analysis of 358 population-based surveys with 1·9 million participants. Lancet..

[56] Narimani M, Esmaeilzadeh S, Azevedo LB, Moradi A, Heidari B, Kashfi-Moghadam M (2019). Association between weight status and executive function in young adults. Medicina..

[57] Stamatakis E, Ekelund U, Ding D, Hamer M, Bauman AE, Lee IM (2019). Is the time right for quantitative public health guidelines on sitting? A narrative review of sedentary behaviour research paradigms and findings. Br J Sports Med.

[58] Chastin SFM, Egerton T, Leask C, Stamatakis E (2015). Meta-analysis of the relationship between breaks in sedentary behavior and cardiometabolic health. Obesity..

[59] Reis M (2015). Uma análise da transição dos jovens para o primeiro emprego no Brasil. Rev Bras Econ.

